# HIV/AIDS prevalence and behaviour in drug users and pregnant women in Kashgar Prefecture: Case report

**DOI:** 10.1186/1477-7517-3-7

**Published:** 2006-02-13

**Authors:** Ni Mingjian, KM Wheeler, J Cheng, Dong Yonghai, W Chen, C Fitzwarryne, J Wang

**Affiliations:** 1 Xinjiang Centre for HIV/AIDS Prevention and Control, 48 Beijing South Road, Urumqi, Xinjiang Uygur Autonomous Region, People's Republic of China; 2 Xinjiang HIV/AIDS Prevention and Care Project, Melbourne Development Institute, 48 Beijing South Road, Urumqi, Xinjiang Uygur Autonomous Region, People's Republic of China

## Abstract

Second Generation sub-population HIV Surveillance was undertaken in Kashgar City and Shache County, Xinjiang, Peoples Republic of China between December 2003 and January 2004, targeting injecting and mixed method drug users and pregnant and postnatal women. The study aimed to determine the extent to which the epidemic is shifting from a concentrated stage to a more generalised epidemic. One hundred and forty two (142) exclusively injecting drug users (66) and mixed method drug users (injecting and non-injecting-76) participated in this survey. Eight hundred and two (802) pregnant and postnatal women participated in the survey. In Kashgar City and Shache County the serum prevalence of HIV amongst injecting drug users was 56.06%, for mixed method drug users 48.68% and 0.38% in pregnant women. In Shache County HIV infection rates were significantly lower in drug user groups and amongst pregnant and post-natal women, at 2.22% and 0% respectively. The behavioral survey indicated that 15% of injecting drug users have shared needles (however sero prevalence and knowledge in relation to access to clean needles and syringes suggests that this may not reflect the actual situation). Knowledge of prevention of transmission strategies (not sharing needles and condom utilisation) is similar between both groups at 60–70%. However it appears that this knowledge has not significantly impacted on behavior such as needle sharing and condom utilisation. In Kashgar City and Shache County there have been very few interventions to support HIV/AIDS prevention, care and control. The results from this survey will inform future directions and the development and implementation of targeted interventions including targeted information dissemination and harm reduction strategies.

This survey was funded by the Xinjiang HIV/AIDS Prevention and Care Project, a bilateral project jointly implemented by the Government of the People's Republic of China and the Government of Australia.

## Background

The secondary surveillance survey was carried out in Kashgar City and Shache County in December 2003 to January 2004. These two areas are within Kashgar Prefecture. The distance between the two is 220 km. The aim of survey was to determine the potential scope of the HIV/AIDS epidemic in Kashgar and whether there was evidence to suggest that this had changed from a concentrated epidemic to a generalised one. There was a cumulative total of 160 HIV positive people in Kashgar Prefecture in June 2003 [1]. These low numbers were of concern when compared with other parts of Xinjiang (in Yining and Urumqi City where the numbers were over 3000 people in both sites) where injecting drug use was high. The survey was managed by the Xinjiang Regional AIDS Prevention Center at the Center for Disease Control (CDC) in collaboration with the Kashgar Prefecture CDC. The results of the survey will be used to inform the development of targeted interventions to specific populations.

## Methods

The 400 participants in the drug user group were registered and unregistered drug users. Among them 310 were from Kashgar City and 90 from Shache County. Participants were mostly men (87.8%) however 49 women participated (12.3%). In the drug user group 142 participants identified themselves as being injecting or mixed method drug users. Serum samples from 802 antenatal and postnatal women were used for this survey. There were 540 participants from Shache County and 262 were from Kashgar City.

The survey was undertaken using anonymous and unlinked serum sampling and a behavioral survey. Investigators confirmed the number of samples by equal probability clustered random sampling. Stratified equal opportunity random sampling was used based on distribution of potential participants using specific settings (for example closed and open settings for drug users). All participants were aware that they were participating in the survey and signed a consent form to have blood taken.

### Behavioural questionnaires

All participants completed behavioral questionnaires during face-to-face interviews. The questionnaires included information on general health, knowledge about HIV/AIDS, attitudes to people living with HIV/AIDS (PLWHA), beliefs in relation to HIV/AIDS prevention and identified at-risk behaviors for HIV/AIDS transmission. At the same time a 5 ml blood sample was taken for serum analysis.

### Serum analysis

Serum analysis for HIV/AIDS was carried out using two ELISA tests on each blood sample. Where both tests were positive this was considered an HIV positive result. Anti -HIV-1/HIV-2 enzyme immunoassay and anti -HIV- 1/HIV-2 enzyme immunoassay were used.

## Results

### HIV prevalence

In the drug user group the sero-prevalence of HIV/AIDS (24.5%) was significantly higher than in the pregnant and postnatal women group (0.13%) indicating that the epidemic remains predominantly within the drug using community. In the injecting and mixed method group (35% of the drug user group) HIV prevalence was significantly higher (56.06% and 48.68% respectively) than in the non-injecting group (8.91%).

When results are disaggregated by area the seroprevalence of HIV was higher in Kashgar City for drug users than pregnant and postnatal women (30.65% and 0.38% respectively) than in Shache County (2.22% and 0% respectively). HIV seroprevalence in the injecting drug user population for Kashgar City (51.42%) indicates a significant HIV epidemic for this population while in Shache County the number of injecting drug users was too small (2) to draw a conclusion.

In recent years there are more reports of HIV in women. In Xinjiang in 2003 (case reporting data) the prevalence of HIV in women was 2.85% for sexual intercourse as the route of transmission and 4.91% from other routes. Women now represent 13.0% of reported cases in Xinjiang [1,2]. In the drug user group for this surveillance the seroprevalence is 4.87% higher in women compared to men.

Injecting drug users who are women are significantly less affected by HIV than men (33% compared to 50.4% respectively). However, when comparing the non-injecting HIV positive group women have a higher prevalence than men (12.9% and 8.29% respectively).

### Behavioral factors impacting on HIV prevalence

Knowledge in relation to HIV/AIDS transmission routes ranged between 60–70% for both groups however the pregnant and postnatal women group had higher levels of understanding (40–50%) in relation to how HIV/AIDS is not transmitted than the drug user group (20–30%).

One variable affecting knowledge and skills is basic education [3]. However there is little variation in basic education between the groups. Location may have an impact on access to higher level education. In a study in Urumqi City 17% of drug users had education to vocational or university education level [4].

Access to information through health services may be a determinant of the differences in knowledge between the two groups. The majority of the pregnant and postnatal women group had received regular antenatal checks where information is provided (686 or 85.5%).

Knowledge does not appear to translate into skills such as not sharing injecting equipment and using condoms. Needle sharing is regarded as a common practice in the drug using community in Xinjiang and contributes to the transmission amongst injecting drug users [5,6]. In the behavioral survey only 15% of drug users indicated that they had shared needles in the last month and last six months. However, 34.7% of all participants did not know where to get clean syringes and needles and 37% of participants thought that it was difficult to find clean syringes and needles.

Condom use was low in both groups participating in the surveillance. HIV seroprevalence was significantly linked to condom use in drug users (84.2% in those who had never used a condom as compared to 6.5% in those using a condom each time they had sexual contact).

## Discussion

The epidemic in Kashgar City remains concentrated in the drug using population and specifically in the injecting drug using community. HIV prevalence differs between the two sites with higher rates in Kashgar City than in Shache County for drug users. In this survey there are also significantly more injecting drug users in Kashgar City (40.3%) compared to Shache County (2.22%). This indicates behavioral differences and geographic barriers.

There are gender differences. Men who are injecting drug users have higher seroprevalence than women injecting drug users. Women amongst the non-injecting drug using population have a higher HIV seroprevalence than their male counterparts however the actual numbers of women are low.

This may be indicative of women not identifying as injecting drug users or alternatively their contracting HIV through sexual transmission. Women in studies in America who participated in needle and syringe exchange programs were found to be more likely to have IDU sexual partners than men [7]. A larger group of women would have to be surveyed to provide indicative results in relation to the impact of gender.

Reported needle and syringe sharing was 15% as compared to 60% reported in another city in Xinjiang and in other parts of China [5]. However there are many variables affecting perceptions of needle and syringe sharing. These include needle sharing being understood as the provider of the needle or syringe being the "equipment sharer" while the recipient is not [8]; knowledge that needle and syringe sharing is not an allowable practice and therefore was not reported; needle sharing not occurring at each event of drug use; and, a misunderstanding of the behavioral questionnaire or a communication issue between the interviewer and the participant.

For the pregnant and postnatal women group condom use was also very low. Factors such as marital status and presumptive protection related to this, cultural norms related to postnatal sexual relations between couples and the predominant view that condoms are considered to prevent pregnancy influence results for this group.

Levels of knowledge in relation to transmission appear to relate to access to the health system. However, lack of knowledge in relation to how HIV/AIDS is not transmitted raises issues in reducing stigma and discrimination in the community towards people living with HIV/AIDS.

## Conclusion

The findings of this survey indicate several key areas of focus for future interventions. These would aim to increase access to knowledge and skills in the general community and in both groups participating in the study. Targeting strategies and implementation programs are critical. Strategies such as peer education have been successfully used to expand a comprehensive harm reduction program in Yining City [9] and this could be applied more broadly in the context. Expansion of harm reduction programs including needle and syringe exchange and access to condoms for injecting drug users is a priority particularly in Kashgar City.

## Authors' contributions

MN conceived and coordinated the project, designed the baseline and mid-term review, provided oversight to the data collection, and undertook initial analysis; KW oversaw the project as a whole; JC and YD provided the technical support and managed the collection of data, JW and CW provided support to the development of the manuscript; and WC supervised the management of the project. All authors had input to the manuscript and read and approved the final version.
